# Successful bridging therapy with tirabrutinib before ASCT for relapsed primary DLBCL of the CNS complicated with PBC, cirrhosis, and pancytopenia

**DOI:** 10.1016/j.lrr.2022.100331

**Published:** 2022-05-30

**Authors:** Noriharu Nakagawa, Ruiko Yamano, Sayaka Kajikawa, Yukio Kondo, Hirokazu Okumura

**Affiliations:** Department of Hematology, Toyama Prefectural Central Hospital, Toyama, Japan

**Keywords:** Primary DLBCL of the CNS, Tirabrutinib, ASCT, Comorbidity

## Abstract

The optimal therapy for relapsed primary diffuse large B-cell lymphoma (DLBCL) of the central nervous system (CNS) remains unclear. We herein report a case of relapsed primary DLBCL of the CNS complicated with primary biliary cholangitis, cirrhosis, and pancytopenia that was successfully treated with bridging therapy with tirabrutinib before autologous hematopoietic stem cell transplantation (ASCT). Tirabrutinib is well tolerated and effective for relapsed primary DLBCL of the CNS with comorbidities, including cirrhosis and pancytopenia. Tirabrutinib is a promising option as bridging therapy before ASCT.

## Introduction

Primary diffuse large B-cell lymphoma (DLBCL) of the central nervous system (CNS) is DLBCL arising within the brain, spinal cord, leptomeninges or eye. A high-dose methotrexate (HD-MTX)-based regimen is the one most commonly used as induction therapy, and consolidation therapy including whole-brain radiation therapy (WBRT) is conventionally performed. In recent years, autologous hematopoietic stem cell transplantation (ASCT) as consolidation therapy has been performed for DLBCL of the CNS in young patients.

Bruton tyrosine kinase (BTK) inhibitors are expected to be useful for primary DLBCL of the CNS, as the B cell receptor signaling pathway is frequently hyperactivated in this disease [1], [2], [3]. Several studies have reported the effectiveness of ibrutinib, a first-generation BTK inhibitor, for relapsed/refractory primary DLBCL of the CNS [4, 5]. Tirabrutinib is a second-generation BTK inhibitor that has been reported to be effective and tolerated in phase I/II studies from Japan. Tirabrutinib is a promising treatment option for relapsed/refractory primary DLBCL of the CNS [6]. However, the optimal therapy strategy for relapsed/refractory primary DLBCL of the CNS remains unclear, and the prognosis is poor [7]. Salvage treatment is difficult in these patients because of their age and comorbidities.

We herein report a case of relapsed primary DLBCL of the CNS complicated with primary biliary cholangitis (PBC), cirrhosis (Child-Pugh A), and pancytopenia due to hypersplenism successfully treated with bridging therapy with tirabrutinib before ASCT.

## Case report

A 53-year-old woman was referred to our hospital with a chief complaint of dizziness. Magnetic resonance imaging (MRI) detected multiple abnormal lesions of her brain, and primary DLBCL of the CNS was diagnosed by a brain biopsy. She had PBC, cirrhosis (Child-Pugh A), and pancytopenia due to hypersplenism as comorbidity. Eight cycles of HD-MTX with rituximab as induction therapy induced a complete response, and she underwent 11 cycles of HD-MTX as consolidation therapy.

**Fig. 1:**
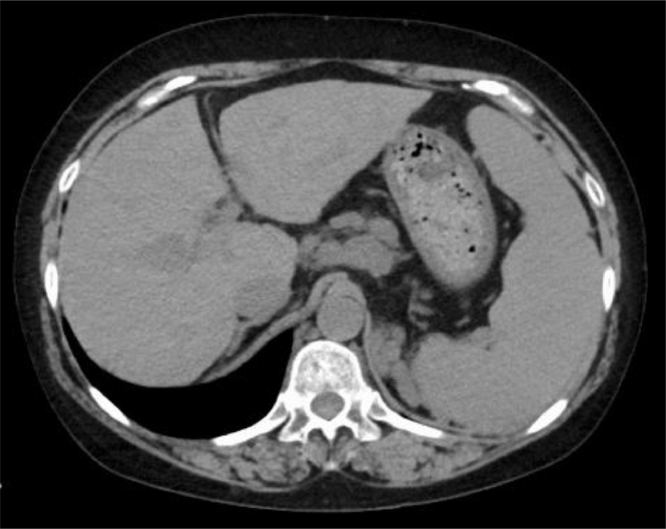
Computed tomography findings showing hepatic morphological changes and splenomegaly.

However, haze in her left eye occurred five months after the end of consolidation therapy, and intraocular lymphoma was diagnosed. MRI detected a new solitary lesion in her brain. Her laboratory data on relapse are shown in Table 1. The values included a neutrophil count of 1.38 × 10^9^/L, hemoglobin level of 98 g/L, and platelet count of 76 × 10^9^/L. Computed tomography showed hepatic morphological changes and splenomegaly (Fig. 1). She received tirabrutinib and intravitreal methotrexate for relapsed disease. Her starting dose of tirabrutinib was 320 mg once a day, and she continued the same dose. Her brain lesion was ameliorated after four weeks of tirabrutinib. She received five intravitreal methotrexate injections, and her vitreous interleukin 10 concentration became undetectable. Progression of cirrhosis or pancytopenia, febrile neutropenia, infections, and rash did not occur during tirabrutinib therapy.

**Table 1:** Laboratory data and vitreous interleukin 6 and 10 concentration on relapse.

White blood cell	2.3	10^9^/L	Total protein	7.7	g/dL	Vitreous interleukin-10	1975	pg/mL
Neutrophil	60	%	Albumin	3.9	g/dL	Vitreous interleukin-6	54	pg/mL
Eosinophil	1.3	%	Aspartate aminotransferase	67	U/L			
Lymphocyte	30.5	%	Alanine aminotransferase	49	U/L			
Red blood cell	3.57		Alkaline phosphatase	100	U/L			
Hemoblobin	98	g/L	Lactate deydrogenase	193	U/L			
Hematocrit	30.5	%	Creatine kinase	56	U/L			
Reticulocyte	44.3	10^9^/L	Glutamyl transpeptidase	88	U/L			
Platelet	76	10^9^/L	Amylase	142	U/L			
Immature platelet fraction	3.1	%	Total bilirubin	1	mg/dL			
			Total cholesterol	153	mg/dL			
Prothrombin time (PT)	13.3	*sec*.	High density lipoprotein cholesterol	50	mg/dL			
PT-international normalized ratio	1.13		Triglyceride	123	mg/dL			
Activated partial thromboplastin time	29.3	*sec*.	Blood urea nitrogen	10	mg/dL			
Fibrinogen	235	mg/dL	Creatinine	0.73	mg/dL			
Fibrin degradation product	< 2.5	μg/mL	Uric acid	5.5	mg/dL			
			Sodium	138	mmol/L			
			Potassium	3.5	mmol/L			
			Chloride	107	mmol/L			
			Calcium	8.7	mg/dL			
			C-reactive protein	0.15	mg/dL			
			Soluble interleukin-2 receptor	708	U/mL

Ten weeks after the start of tirabrutinib, 7.7 × 10^6^ CD34⁺ peripheral blood stem cells per kilogram were collected using granulocyte colony-stimulating factor (G-CSF) and plerixafor. She was treated with ASCT following conditioning with busulfan (3.2 mg/kg) on days -6 to -5 and thiotepa (5 mg/kg) on days -4 to -3. Neutrophil engraftment occurred on day 13. Veno-occlusive disease/sinusoidal obstruction syndrome did not occur. No relapse of her lymphoma was noted during the follow-up period of 6 months after ASCT.

## Discussion

In recent years, the pathogenesis of primary DLBCL of the CNS has been clarified; however, the optimal therapeutic strategy for relapsed/refractory primary DLBCL of the CNS remains unclear [8]. There are several issues with the management of primary DLBCL of the CNS. Chemotherapy including conventional cytotoxic agents carries a higher risk of causing complications in patients with comorbidities than in those without comorbidities. Patients with pancytopenia in particular have higher risk of infections and bleeding due to bone marrow suppression than other patients. Although WBRT is used for primary DLBCL of the CNS as a consolidation therapy, to avoid the risk of late-onset neurotoxicity due to WBRT, ASCT is sometimes used as a consolidation therapy instead of WBRT, especially in young patients.

One study reported that HD-MTX remains effective for relapsed primary DLBCL of the CNS in patients who initially respond to methotrexate [9]. As such, salvage therapy with HD-MTX is considered a promising treatment option in these patients. Since our present case experienced recurrence early after consolidation therapy with HD-MTX, we selected tirabrutinib after considering the possibility of HD-MTX resistance to her relapsed lymphoma. High-dose cytarabine regimen is the most used treatment in MTX refractory CNS lymphoma; however, there was concern that the risk of infection and bleeding due to myelosuppression would increase in this patient complicated with PBC, cirrhosis, and pancytopenia.

Intraocular lymphoma was diagnosed at the time of her relapse. A phase Ⅰ/II study of tirabrutinib included 3 patients with intraocular lymphoma, and all of them achieved partial response [6]. On the other hand, the efficacy of combined intravitreal MTX and chemotherapy for intraocular lymphoma was reported [10]. As such, we considered intravitreal MTX in combination with tirabrutinib.

A phase Ⅰ/II study of tirabrutinib reported that the overall response rate was 64%, and the median progression-free survival was 2.9 months [6]. Tirabrutinib was considered to have favorable efficacy in patients with relapsed/refractory primary DLBCL of the CNS. Yoshioka et al. reported that tirabrutinib could be administered via nasogastric tubes to treat elderly patients with primary DLBCL of the CNS [11], suggesting this agent is highly versatile. However, the safety of tirabrutinib is unclear, as there are few reports of cases with comorbidity. The optimal dose of tirabrutinib in patients with cirrhosis is also unknown. In our present case, the dose was reduced to 320 mg daily. The patient's myelosuppression and liver function after starting tirabrutinib were acceptable. We were going to consider increasing the dose if the efficacy of tirabrutinib proved inadequate, but her brain lesion was ameliorated after four weeks of therapy.

One of the problems with administering tirabrutinib monotherapy for relapsed/refractory primary DLBCL of the CNS is that the duration of the response is inadequate. For this reason, tirabrutinib as a bridging therapy before ASCT is effective, since ASCT may be able to lengthen the progression-free survival for patients with relapsed/refractory primary DLBCL of the CNS. However, the influence of tirabrutinib on peripheral blood stem cell harvesting remains unclear, although we were able to collect adequate peripheral blood stem cells in our patient using G-CSF and plerixafor. Further studies of tirabrutinib as maintenance therapy after ASCT are also warranted.

We herein report a case of relapsed primary DLBCL of the CNS complicated with PBC, cirrhosis, and pancytopenia due to hypersplenism successfully treated with bridging therapy with tirabrutinib before ASCT. Tirabrutinib is well tolerated and effective for relapsed primary DLBCL of the CNS with comorbidities, including cirrhosis and pancytopenia. Tirabrutinib is a promising option as bridging therapy before ASCT. Further studies are required to determine the optimal therapeutic strategy for relapsed/refractory primary DLBCL of the CNS.

## Declaration of Competing Interest

The authors declare no conflicts of interest in association with the present study.
